# Central blood pressure and arterial stiffness among ultramarathon runners across the lifespan

**DOI:** 10.1007/s00421-025-05924-w

**Published:** 2025-07-30

**Authors:** Joseph D. Vondrasek, Soolim Jeong, Omar B. El-Kurd, Braxton A. Linder, Nina L. Stute, Christin Domeier, Thomas G. Bissen, James R. Bagley, Austin T. Robinson, Matthew C. Babcock, Gregory J. Grosicki, Joseph C. Watso

**Affiliations:** 1 Cardiovascular and Applied Physiology Laboratory, Department of Health, Nutrition, and Food Sciences, Florida State University, Tallahassee, FL, USA; 2 Institute of Sports Sciences and Medicine, Department of Health, Nutrition, and Food Sciences, Florida State University, 120 Convocation Way, Tallahassee, FL 32306, USA; 3 Neurovascular Physiology Laboratory, School of Kinesiology, Auburn University, Auburn, AL, USA; 4 Muscle Physiology Laboratory, Department of Kinesiology, San Francisco State University, San Francisco, CA, USA; 5 Neurovascular Physiology Laboratory, School of Public Health, Indiana University, Bloomington, IN, USA; 6 Division of Geriatric Medicine, University of Colorado Denver, Anschutz Medical Campus, Denver, CO, USA; 7 Biodynamics and Human Performance Center, Department of Health Science and Kinesiology, Georgia Southern University–Armstrong Campus, Savannah, GA, USA; 8 Institute for Successful Longevity, Department of Psychology, Florida State University, Tallahassee, FL, USA

**Keywords:** Aging, Ultramarathon, Blood pressure, Central stiffness, Endurance exercise

## Abstract

**Aim:**

Regular exercise is beneficial, but more exercise may not always benefit cardiovascular health (extreme exercise hypothesis). This is concerning, because ultramarathon participation is growing, but previous work on cardiovascular health among ultramarathon runners is equivocal. Prior work has not examined this population’s age-related differences in prognostic cardiovascular health metrics.

**Purpose:**

Measure brachial and central BP and arterial stiffness (carotid-to-femoral pulse wave velocity [cfPWV]) among ultramarathon runners.

**Methods:**

We measured supine BP and cfPWV (SphygmoCor-XCEL) among 71 athletes (16 female/55 male; body mass index: 22.6 ± 1.8 kg/m^2^) 1–3 days before they competed in the 161-km Western States Endurance Run. We present data as mean ± SD. We analyzed the relation between age and central BP, brachial BP, and cfPWV with nonlinear (quadratic) regression.

**Results:**

There was a significant curvilinear relation between age (46 ± 10; range 26–69 years) and central (116 ± 8 mmHg, R^2^ = 0.18, *P* = 0.02) but not brachial (129 ± 9 mmHg, R^2^ = 0.06, *P* = 0.11) systolic BP. There was a significant relation between age and central (79 ± 7 mmHg, *R*^2^ = 0.24, *P* < 0.001) and brachial (78 ± 7 mmHg, *R*^2^ = 0.23, *P* < 0.001) diastolic BP. There was a significant (R^2^ = 0.31, *P* = 0.02) curvilinear relation between age and cfPWV (6.5 ± 1.0m/s). Average brachial systolic BP differed by + 1.4 mmHg/decade, and 86% of athletes had a cfPWV below age-predicted (mean difference: − 0.9 ± 1.0 m/s).

**Conclusions:**

These are among the first data to characterize central BP and arterial stiffness across a wide age range of ultramarathon runners. Further work is needed to determine the longitudinal changes associated with training for and competing in ultramarathons.

## Background

Cardiovascular disease (CVD) is the leading cause of death in the United States and worldwide (Martin et al. 2024), with age being the strongest risk factor (Martin et al. 2024). Increased CVD risk with advancing age is partly attributable to elevated blood pressure (BP) (Chobanian et al. 2003) and central arterial stiffening (Boutouyrie et al. 2021; Reference Value Collaboration 2010). While age is not modifiable, increasing exercise can slow cardiovascular aging (Seals et al. 2019). Previous research has demonstrated an inverse dose–response relation between moderate-to-vigorous intensity exercise time and all-cause mortality risk, incidence of CVD, and CVD mortality (Kraus et al. 2019; Lear et al. 2017). Interestingly, some studies have reported aerobically trained adults had higher blood pressure (BP) than adults who were sedentary (Laurent et al. 2011), but the majority of studies report that aerobic exercise training is associated with BP reduction (Edwards et al. 2023). Aerobically trained adults have lower arterial stiffness compared with untrained adults in cross-sectional studies (Tanaka et al. 1998; Vaitkevicius et al. 1993; Laurent et al. 2011). In general, increasing exercise volume is considered beneficial for health. However, some evidence suggests that more exercise may not always be beneficial and could even increase CVD risk (Franklin et al. 2020).

The Extreme Exercise Hypothesis suggests that the dose–response relation between exercise and cardiovascular health benefits is a U- or reverse J-shaped curve, such that excessive exercise volume will increase CVD risk (e.g., increase risk of atrial fibrillation or myocardial fibrosis) (Eijsvogels et al. 2018; Andersen et al. 2013; Breuckmann et al. 2009; [Bibr R36][Bibr R58]). Concerns about “extreme” exercise volumes and cardiovascular health are pertinent given the rising participation in ultra-endurance events (Scheer 2019). Participation in ultramarathon running (i.e., any running event longer than a 42.2 km marathon) has grown 1676% in the past 23 years, and the number of races and participants in those races has increased exponentially over the past ~30 years (Ronto 2024). Ultramarathon competition requires training volumes three-to-five times higher than the current guidelines of at least 150 min per week of moderate-intensity exercise (Belinchón-deMiguel et al. 2021; Thuany et al. 2022). Although ultramarathon runners still represent a relatively small subset of the overall running community, the growing athlete population results in the opportunity for researchers to investigate extreme exercisers (Babcock and Robinson 2024). Also, studying the effects of extreme exercise may inform exercise guidelines for achieving optimal cardiovascular health.

Previous research on extreme exercisers and cardiovascular health produced equivocal findings, and few have examined wide age ranges. In a survey-based study, only 9% of ultra-endurance runners reported hypertension, and 5% had established CVD (Shah et al. 2021). Some studies report elevated BP (Radtke et al. 2014; Belinchón-deMiguel et al. 2019; Ranadive et al. 2024) and lower (worse) large artery compliance (the inverse of artery stiffness) among experienced ultra-distance runners compared with recreationally active participants (Burr et al. 2014). However, another group found no difference in brachial-ankle pulse wave velocity between male marathon runners, ultramarathon runners, and active participants (Radtke et al. 2014). Overall, these mixed findings highlight the need for more research among the quickly growing ultramarathon population.

Therefore, we characterized prognostic metrics of cardiovascular health—central BP, peripheral BP, and arterial stiffness—among a wide age range of ultramarathon runners. To further contextualize our results, we hypothesized that age-related differences in BP and central arterial stiffness would be favorable compared with published data (Ahmadi-Abhari et al. 2017; Tanaka et al. 2000; Vaitkevicius et al. 1993; Climstein et al. 2023).

## Methods

### Ethical approval

This study protocol and informed consent were approved by the Institutional Review Board of Georgia Southern University (H23253). The protocol conformed with the Declaration of Helsinki except for trial preregistration, because participants were not assigned to an intervention. Participants were fully informed of all the experimental procedures and the potential risks of participation before providing written and informed consent. The data collected in this project are part of a larger project with a separate hypothesis that was recently published (Babcock et al. 2024).

#### Study overview:

Athletes who qualified for the 2023 *Western States Endurance Run* (WSER) were contacted via email ~ 2 months before race day. Interested individuals who gave consent were sent pre-race surveys. Participants completed pre-race cardiovascular assessments 1–3 days before race day. Athletes were asked to refrain from strenuous exercise, caffeine, tobacco, and alcohol, and from consuming calorie-containing food or drinks for at least 3 h before testing (Muntner et al. 2019). All cardiovascular measures were obtained in a canopy tent with space heaters and fans to control temperature.

#### Participants:

Inclusion criteria were ≥ 18 years old, qualification for and intent to participate in the 2023 WSER, and willingness to comply with study procedures: completing demographic, medical history, lifestyle questionnaires, and pre-race cardiovascular assessments.

#### Pre-race survey.

The pre-race survey asked for demographics, training history, ultramarathon experience, medical history, and medication use in the last three months.

#### Cardiovascular assessment:

We measured brachial BP with an automated brachial cuff (SphygmoCor-XCEL, AtCor Medical, Naperville, USA) after at least 5 min of quiet supine rest. Next, we performed pulse wave analysis (PWA) and then measured carotid-to-femoral pulse wave velocity (cfPWV), an index of central arterial stiffness. We used the same procedures we previously described (Babcock et al. 2024).

#### Pulse wave analysis:

The brachial cuff automatically inflated to a sub-systolic pressure, and brachial BP waveforms were acquired. Waveforms were transformed using a validated generalized transfer function to estimate an aortic pressure waveform (Butlin et al. 2012; Pauca et al. 2001). We derived central BP, central pulse pressure (PP), central augmentation pressure (AP), central augmentation index (AIx), and AIx normalized to a heart rate of 75 bpm (AIx75) from the transfer function. The PP is the difference between central systolic and diastolic BP. AP is the difference between the first and second systolic peaks and represents the change in systolic pressure due to the arrival of the returning pressure wave. AIx (%) is AP expressed as a percentage of PP (AP/PP*100). AIx75 is AIx normalized to a heart rate of 75 bpm because of the known effect HR has on AIx (Wilkinson et al. 2000). Measures were taken in duplicate or until two measures had a systolic BP difference ≤ 5 mmHg; the two closest measures were averaged. We calculated systolic BP amplification as: brachial systolic BP–central systolic BP (Herbert et al. 2014). To contextualize our results, we compared our findings to published data on age-related differences in systolic BP (Chobanian et al. 2003; Gurven et al. 2012; Intersalt Cooperative Research Group 1988; Climstein et al. 2023).

#### Pulse wave velocity:

Trained investigators (JDV, GJG, and MCB) obtained signals to determine central arterial stiffness with cfPWV (SphygmoCor-XCEL, AtCor Medical, Naperville, USA) via applanation tonometry using the subtraction method. We placed a tonometer on the site of the strongest carotid pulse while a thigh cuff simultaneously inflated to a sub-systolic pressure. cfPWV was calculated as cfPWV = D/PTT, where D is the arterial path length, and PTT is the pulse transit time. We measured D with a non-elastic tape measure as the linear distance from the suprasternal notch to the top of the cuff at the superficial femoral artery, subtracting the distance from the suprasternal notch to the common carotid artery palpation site. PTT was measured with a proprietary algorithm that automatically calculated the time between the foot of the proximal (carotid) and distal (femoral) pressure waveforms (Butlin et al. 2013). Measures were taken in duplicate or until two measures differed by ≤ 0.5 m/s; the two closest measures were averaged (mean absolute difference: 0.22 m/s). To contextualize our results, we compared our findings with published data on age-related differences in cfPWV at a population level (Reference Value Collaboration 2010).

#### Data and statistical analysis:

We assessed normality via Shapiro–Wilk tests and visual inspection of quantile-to-quantile (QQ) plots. We present normally distributed data (Shapiro–Wilk test; *P* > 0.05) as mean ± SD and non-normally distributed (Shapiro–Wilk test; *P* ≤ 0.05) data as median [IQR]. We present categorical variables from the pre-race survey as count (%). Statistical significance was set a priori at *α* < 0.05.

For regression analysis, we used scatterplots and examined residual distribution normality to determine the type of regression analysis (*e.g.*, linear vs. quadratic). We assessed the relation between age and brachial systolic and diastolic BP, age and central systolic and diastolic BP, and age and BP amplification with curvilinear regression. Previous research suggests that there would be a curvilinear relation between age and both AIx and AIx75, because past work found that pressure augmentation increases faster at younger ages but plateaus after age ~ 60 (Tomiyama et al. 2014). We completed curvilinear regression, but this model was not significant, and the data appeared to follow a linear pattern. Thus, we used a linear regression model to assess the relation between age and AIx and AIx75. We interpreted coefficients of determination (*R*^2^) as *negligible* (0.0 to < 0.09), *low* (0.09 to < 0.25), *moderate* (0.25 to < 0.49), *high* (0.49 to < 0.81), and *very high* (0.81 to 1.0) (Mukaka 2012; Hinkle et al. 2003). We first quantified the relation between age and cfPWV with curvilinear regression, but the residuals were not normal, so we transformed cfPWV using log_10_ and performed curvilinear regression.

We used results from the Reference Values for Arterial Stiffness Collaboration to help contextualize age-related differences in cfPWV in this WSER cohort. The Reference Values for Arterial Stiffness Collaboration included central arterial stiffness measures among 16,000 + participants, 15–97 years old (Reference Value Collaboration 2010). We used the age- and BP-based prediction equations to provide context for our findings. We log-transformed (log_10_) age-predicted values for each prediction equation, so the predicted values could be compared with the data we collected in this investigation.

We measured the direction and strength of relations between training volume leading up to the 2023 WSER and baseline cardiovascular measures (brachial BP and cfPWV) with Spearman’s ρ. We used partial ρ correlation to assess the strength and direction of relations between years of running experience and brachial BP and cfPWV while controlling for age. We interpreted ρ as *negligible* (0.0 to < 0.30), *low* (0.30 to < 0.50), *moderate* (0.50 to < 0.70), *high* (0.70 to < 0.90), and *very high* (0.90 to 1.0) (Mukaka 2012; Hinkle et al. 2003).

We categorized brachial BP status in this sample based on the current American College of Cardiology/American Heart Association clinical guidelines, despite measurement in the supine position, consistent with pulse wave analysis and cfPWV assessments (Whelton et al. 2018; Flack and Adekola 2020). Brachial BP was categorized as follows: normal (systolic BP < 120 mmHg AND diastolic BP < 80 mmHg), elevated (systolic BP 120–129 mmHg AND diastolic BP < 80 mmHg), stage 1 hypertension (systolic BP 130–139 mmHg OR diastolic BP 80–89 mmHg), and stage 2 hypertension (systolic BP 140–180 mmHg OR diastolic BP 90–120 mmHg).

## Results

### Participants:

A total of 75 athletes completed the pre-race survey or cardiovascular testing. 72/75 completed both the survey and the cardiovascular testing. We removed one male athlete with exceptionally high resting brachial BP (*i.e.,* > 180/90 mmHg) who tested positive for COVID-19 via Polymerase Chain Reaction test 2 days after completing the WSER (4 days after pre-race cardiovascular testing). Thus, we included 71 participants in the final analysis (55 male, 16 female, ~ 22% female). 80% of the participants qualified via the WSER lottery. Participant characteristics, demographics, and training history are shown in Table 1.

### Survey:

**Fig. 1:**
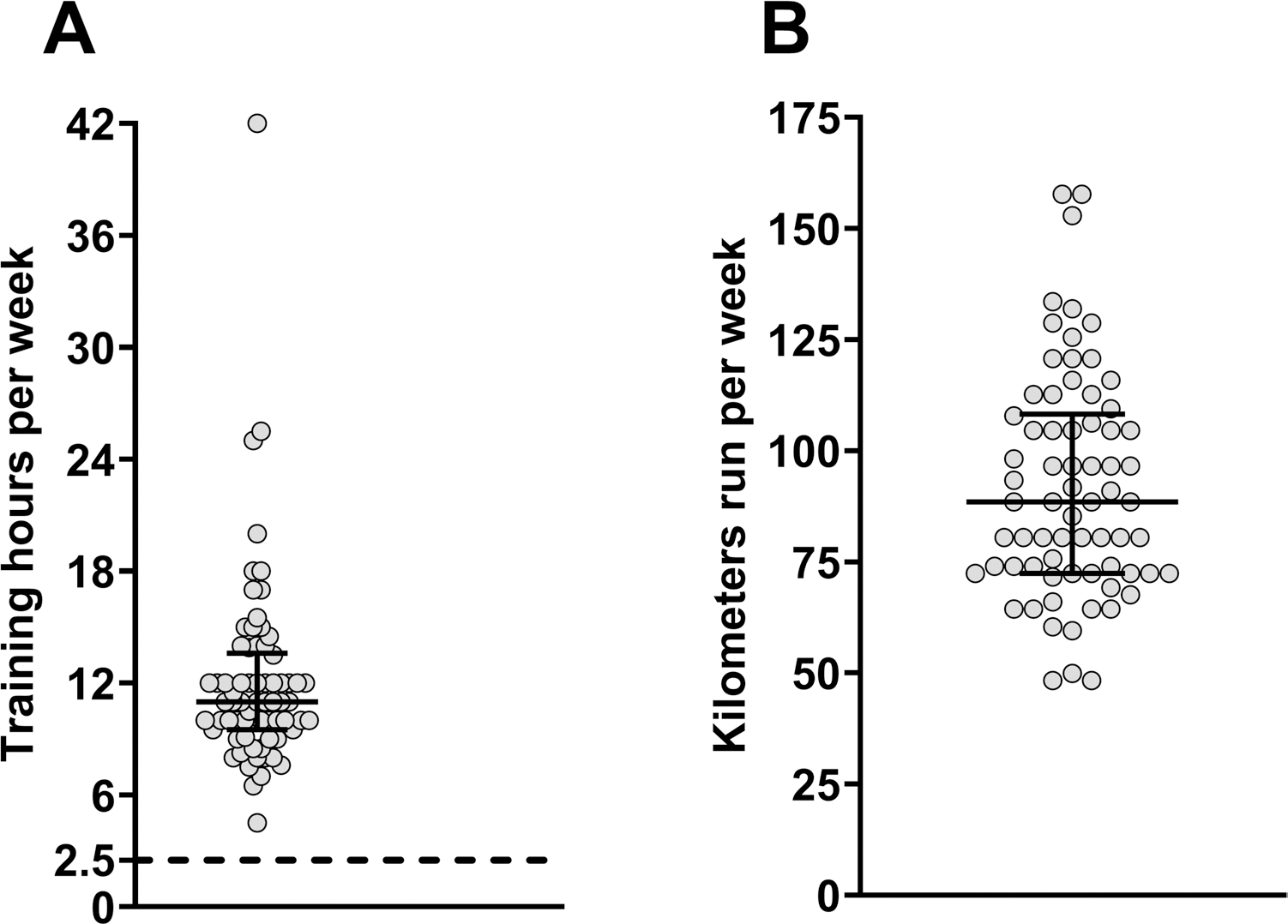
Participant response to the pre-race survey for weekly training time (**A)** and distance (**B**) in the three months leading up to the WSER. Each circle represents one participant. The dotted line in Panel A represents the current recommendation (2.5 h) for weekly moderate-intensity exercise. The error bars around the median represent IQR for both panels. *n* = 70 for each panel

We present weekly training time in hours per week (**Panel A**) and kilometers run per week (**Panel B**) during the 3 months leading up to the WSER in Fig. 1. 75% of the sample had not previously started the WSER, and 80% had not previously finished the WSER. All athletes completed at least one ultramarathon in the previous year (required for WSER entry), and 42% of the sample completed ≥ 2 ultramarathons. We present the participants’ medical history in Table 2.

**Fig. 2:**
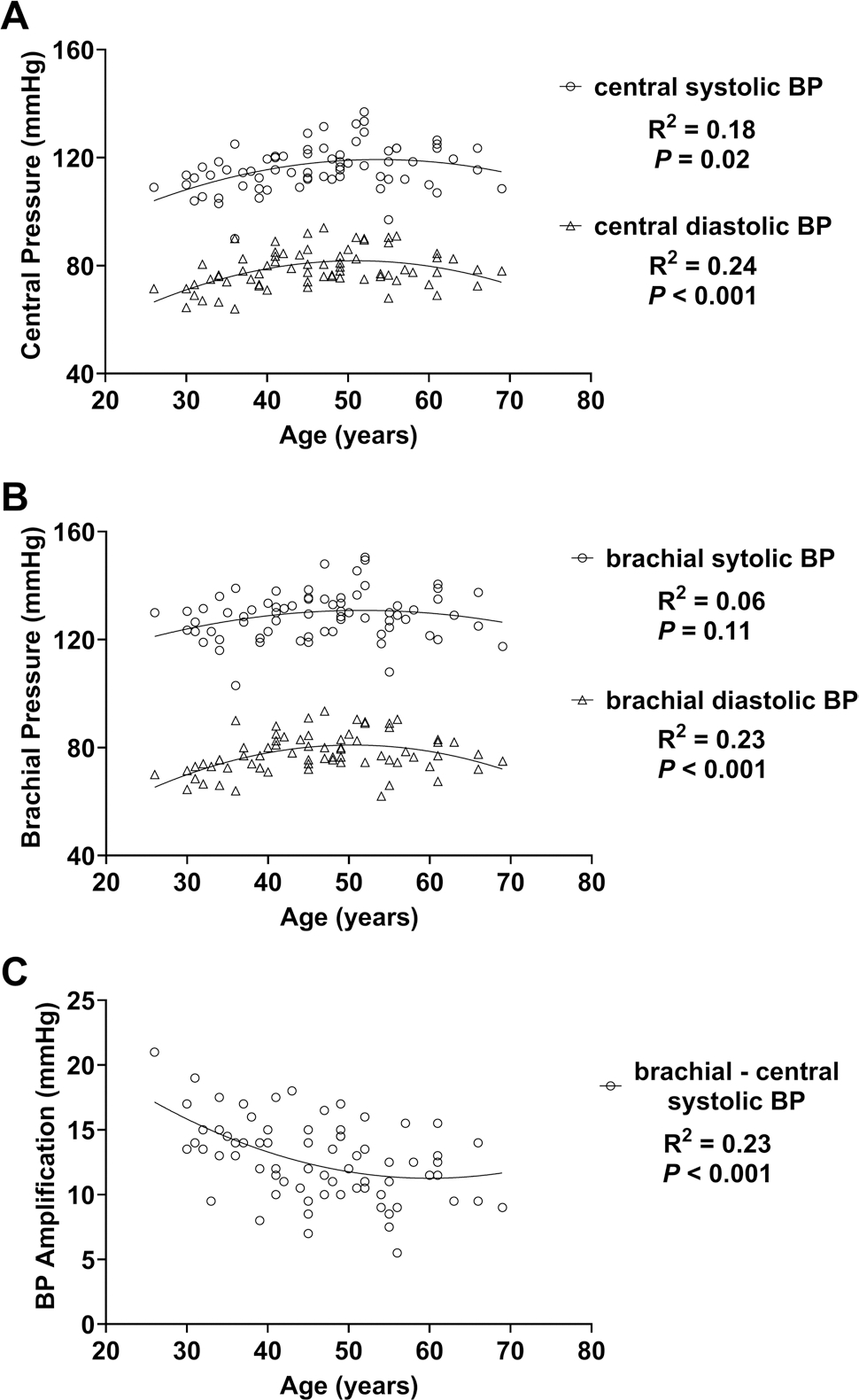
Based on curvilinear regression, there was a significant *low* curvilinear relation between age and central systolic (**A)** and diastolic (**A)** BP. There was no relation between age and brachial systolic (**B)** and diastolic (**B)** BP. There was a significant *low* curvilinear relation between age and BP amplification. *BP* blood pressure. BP amplification = brachial systolic BP–central systolic BP

Based on curvilinear (quadratic) regression, there was a significant *low* curvilinear relation between age and central systolic BP (Fig. 2, **Panel A**), and between age and central diastolic BP (Fig. 2, **Panel A**). Based on curvilinear (quadratic) regression, there was no curvilinear relation between age and brachial systolic BP (Fig. 2, **Panel B**) and a significant *low* curvilinear relation between age and brachial diastolic BP (Fig. 2, **Panel B**). Based on curvilinear (quadratic) regression, there was a significant *low* curvilinear relation between age and BP amplification (Fig. 2, **Panel C**).

**Fig. 3:**
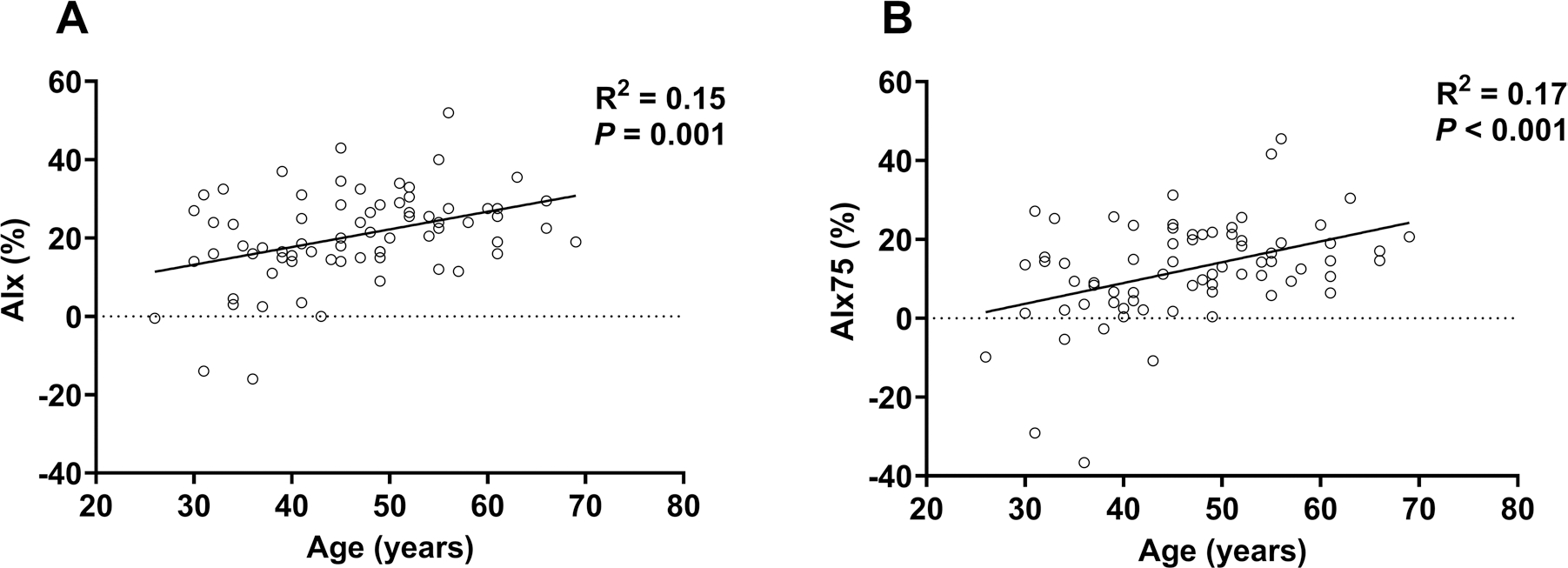
Based on linear regression, there was a significant *negligible* linear relation between age and AIx (**A)** and AIx75 (**B)**. *AIx* augmentation index, *AIx75* augmentation index normalized to a heart rate of 75 beats per minute

Based on linear regression, there was a significant *negligible* linear relation between age and AIx (Fig. 3, **Panel A**) and between age and AIx75 (Fig. 3, **Panel B**).

### Pulse wave velocity:

**Fig. 4:**
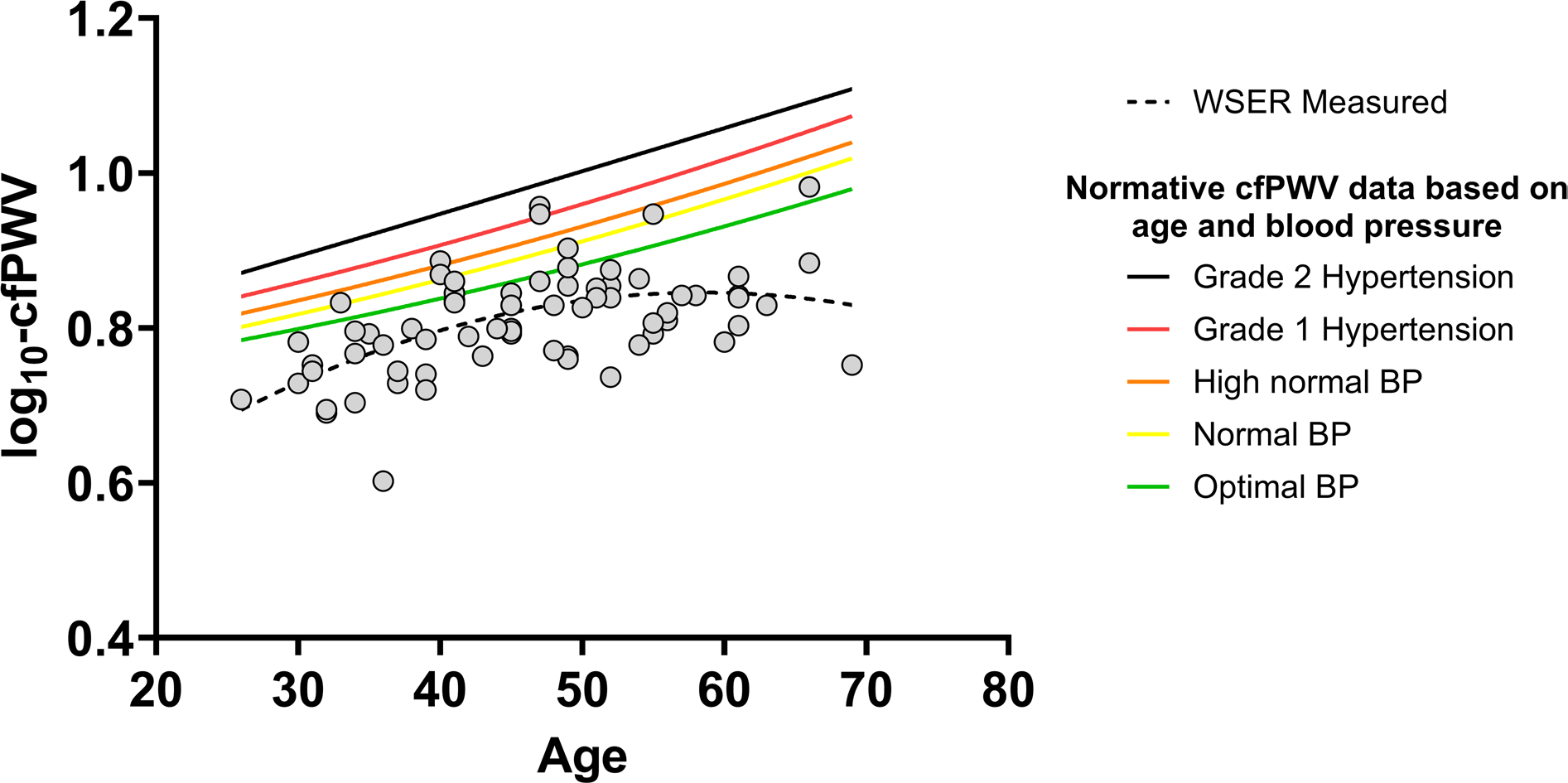
There was a significant *moderate* curvilinear relation between age and carotid-to-femoral pulse wave velocity (cfPWV). Each open circle represents one participant in this study. The dotted line represented the curvilinear regression line for this sample. Each solid line represents age-predicted cfPWV based on the blood pressure (BP) category reported in the reference values for arterial stiffness collaboration. The BP categories used in the references values are optimal BP: systolic < 120 AND diastolic < 80 mmHg; normal BP: systolic 120–129 mmHg OR diastolic 80–84 mmHg; high normal BP: systolic 130–139 mmHg OR diastolic 85–89 mmHg; grade 1 hypertension: systolic 140–159 mmHg OR diastolic 90–99 mmHg; grade 2 hypertension: systolic ≥ 160 OR diastolic ≥ 100 mmHg

We did not measure cfPWV for one participant, and we removed one more participant, because her cfPWV was ~ four standard deviations below the mean and lower than any cfPWV values we had seen reported (measure #1: 2.2 m/s, measure #2: 2.2 m/s). Thus, we included 69 athletes in the final cfPWV analysis. Based on curvilinear regression, there was a *moderate* relation between age and cfPWV (R^2^ = 0.31, *P* = 0.02, Fig. 4). The average cfPWV was 6.5 ± 1.0 m/s and 59/69 (86%) WSER athletes had a cfPWV below their age-predicted value based on normative data for “optimal” BP (< 120/80) (Reference Value Collaboration 2010). On average, cfPWV was below age-predicted by 0.9 ± 1.0 m/s. There were no associations between cfPWV and training distance (ρ = −0.16, *P* = 0.19) and training time (ρ = −0.17, *P* = 0.16); there was a non-significant association between cfPWV and years of running experience when controlling for age (ρ = 0.19, *P* = 0.25).

We used simple linear regression to determine age-related differences in brachial systolic BP to compare our findings to previous findings. Based on this regression, the difference in systolic BP per decade was + 1.4 mmHg/decade (systolic BP = 122.60216 + 0.1368115*Age). Based on current seated brachial BP guidelines, 9/71 (12.7%) athletes had normal BP, 19/71 (26.8%) athletes had elevated BP, 34/71 (47.9%) athletes had Stage 1 hypertension, and 9/71 (12.7%) athletes had Stage 2 hypertension based on their supine BP values. There were no associations between systolic BP and training distance (*ρ* = − 0.11, *P* = 0.39), training time (*ρ* = − 0.02, *P* = 0.86), or running history (*ρ* = 0.14, *P* = 0.25).

## Discussion

### Primary findings:

We aimed to characterize central BP and arterial stiffness among ultramarathon runners and examine age-related differences in this unique population. Our primary findings were: (1) there was a significant low relation between age and central but not brachial systolic BP among WSER athletes, (2) the age-related difference in brachial systolic BP among WSER athletes was + 1.4 mmHg per decade, (3) there was a significant *moderate* association between age and cfPWV, and 4) 86% of the WSER athletes had cfPWV values below (*i.e.,* better than) their age-predicted value.

### Age and central blood pressure:

There was a significant relation between age and central systolic and diastolic BP (Fig. 2). Previous work in a large cohort (*n* = 45,436) found that central systolic and diastolic BP tend to increase with age, and the increase with age is more pronounced among those with CVD risk factors like hypertension (Herbert et al. 2014). To our knowledge, we are the first to measure central BP across a wide age range of ultramarathon runners, so more work is needed in this growing population. Central BP in this WSER cohort was comparable (central BP: 116/79) to one ultra-endurance athlete cohort (central BP: 111/87) but was higher on average than another similar cohort (central BP: 106/72) (Bachman et al. 2021; Knez et al. 2008). Central BP is considered a key cardiovascular health marker, because aortic pressure better reflects the pressure that target organs (e.g., brain, kidneys) experience (McEniery et al. 2014). The potential effects of body position, travel, and altitude could have influenced central BP. Considering these limitations, more work is needed to understand typical central BP in this unique population.

### Age and pulse wave velocity:

We found a significant association between age and cfPWV (Fig. 4). The increase in stiffness with age is supported by the curvilinear decrease in BP amplification with age we observed (Fig. 2), which reflects central arterial stiffening (Herbert et al. 2014). Based on normative values in participants with ‘optimal’ BP (< 120 AND 80 mmHg), 86% of this WSER sample had cfPWV below their age-predicted values (Reference Value Collaboration 2010). Increased arterial stiffness is a hallmark of cardiovascular aging (Chirinos et al. 2019), and the positive association between age and arterial stiffness has been previously demonstrated (Reference Value Collaboration 2010; Vaitkevicius et al. 1993). Previous work comparing physically active participants and ultra-endurance athletes found that arterial compliance was lower (worse), brachial-ankle pulse wave velocity was not different, and cfPWV was lower (better) in the ultra-endurance athletes (Bachman et al. 2021; Burr et al. 2014; Radtke et al. 2014). These mixed findings highlight the lack of homogeneity of cardiovascular adaptation to extended-duration aerobic training. Our findings align with Bachman and colleagues, because many athletes had lower arterial stiffness than predicted (Bachman et al. 2021). Our findings support previous findings showing the slowing of arterial stiffening with regular aerobic exercise (Ahmadi-Abhari et al. 2017; Tanaka et al. 1998; Vaitkevicius et al. 1993), even in the context of very high training loads. Moreover, our results support recent work that found that age-related arterial stiffening (brachial-ankle pulse wave velocity) is slower among adults with higher cardiorespiratory fitness (Jae et al. 2024).

### Age and augmentation index:

We did not find a curvilinear relation between age and AIx or AIx75, but we did find a significant linear relation between age and AIx or age and AIx75 (Fig. 3). This was surprising, because the previous work has noted a curvilinear pattern of change (Tomiyama et al. 2014). A previous investigation comparing central hemodynamics and pressure augmentation between ultra-endurance triathletes and recreationally active control participants found that radial AIx was not different between groups (Knez et al. 2008). In contrast, another study found that AIx was lower in ultra-endurance athletes (Edwards and Lang 2005); the discrepancy was attributed to the different fitness levels of control groups. Our data did appear to follow the expected pattern of increase at the younger ages, with a leveling around 60 years old, but a curvilinear model was not statistically significant (*P* > 0.21). The lack of a curvilinear relation could be explained by the lack of a significant association between age and PP (*ρ* = 0.12, *P* = 0.34), and the *low* positive association between age and AP (*ρ* = 0.35, *P* = 0.003). Mathematically, if AP and PP both increase linearly, an expected change with age in the general population, the resulting ratio (AIx = AP/PP) creates a curvilinear relation (Namasivayam et al. 2010). AP is partly explained by the arrival of the returning pressure wave after or during the incident pressure wave. The arrival is influenced by arterial stiffness, which we found to be lower than age-predicted among many athletes. Thus, it could be that lower arterial stiffness is driving the lack of a curvilinear association between age and AIx. To our knowledge, we are the first to examine age-related differences in central pressure augmentation in the ultramarathon runner population. Thus, further research is needed to understand the age-related differences in pressure augmentation among ultra-endurance athletes.

### Brachial blood pressure:

Our finding is similar to other investigations that found elevated resting brachial BP among ultra-endurance athletes compared to control participants (Radtke et al. 2014), among finishers and non-finishers of an ultramarathon (Belinchón-deMiguel et al. 2019), and among experienced runners before a 50-km race (Ranadive et al. 2024). We are not the first to find elevated brachial BP in this population, but we use caution when classifying these BP measures according to clinical guidelines, because our measures were not taken in a clinical setting. External factors such as travel or general excitement about the WSER may have contributed to these findings. Some studies suggest that brachial BP increases in the supine position (Netea et al. 2003; T-M et al. 2008), but others have found that BP was higher while seated compared to supine (Privšek et al. 2018). Some (Rhodes et al. 2011), but not all (Salvi et al. 2022) evidence suggests that the altitude of Olympic Valley could contribute to this finding (Olympic Valley, CA: ~ 1890m). It is unclear how each of these factors influenced BP. Interestingly, we did not find an association between training volume and brachial systolic BP, and the prevalence of hypertension reported in the health history survey was incongruent with our brachial BP results, because few participants reported having diagnosed hypertension, while many participants had elevated BP measures in this study. Because we did not measure these results in a clinical setting, and multiple external factors could have influenced BP, the BP classifications should not be treated as a clinical diagnosis. However, the BP classifications provided context to our findings, and we are not the first to find elevated brachial BP in this population. Considering these points, more work is needed to understand typical brachial BP in this rapidly growing population.

There was a significant relation between age and central systolic and diastolic BP and between age and brachial diastolic, but not brachial systolic BP (Fig. 2). Interestingly, the Framingham Heart Study and the Baltimore Longitudinal Study on Aging reported a linear association between age and brachial systolic BP among the general population (Franklin et al. 1997; Vaitkevicius et al. 1993). Also, the relation between age and diastolic BP we observed had been previously characterized (Franklin et al. 1997). The decrease in diastolic BP and subsequent widening of pulse pressure with age is partly attributable to arterial stiffening with age (Izzo 2004); we observed a significant positive association between age and central arterial stiffness.

The difference in systolic BP per decade was + 1.4 mmHg in this WSER cohort. Investigations in the general population have reported a wide range of systolic BP differences with age from 0.9 to 13.3 mmHg/decade (Chobanian et al. 2003; Gurven et al. 2012; Intersalt Cooperative Research Group 1988; Climstein et al. 2023), with many reporting a slope steeper than what we found in this WSER cohort. One group studied the Tsimane people, a hunter-gatherer/forager/farmer community, found slow increases in BP (female: 2.86 mmHg/decade, males: 0.91 mmHg/decade, Tsimane average: 1.9 mmHg/decade), which could be due to higher physical activity and fewer calories coming from highly palatable calorically dense foods (Gurven et al. 2012). Another group investigated BP among World Masters Games athletes (Climstein et al. 2023). The study was limited by survey-reported BP, but the average difference per decade was + 1.8 mmHg among 2,793 athletes < 30 to 80–89 years old (Climstein et al. 2023). The relatively small difference in systolic BP across the age range of WSER athletes suggests maintenance of BP across the lifespan, which reflects lower CVD risk. The protective effects of aerobic exercise could be driving this difference (Edwards et al. 2023). Alternatively, the smaller systolic BP difference could be partly explained by the age range of our study not including many adults older than 65 (3/71 over 65 years), a population known to have a higher (~ 75%) prevalence of hypertension than adults 35 to 44 (~ 40%) (Martin et al. 2024). If the maximum age for ultramarathon participants increases, this question could be answered in future studies. Further research is needed to understand the prospective changes in BP across the lifespan of current and former ultramarathon runners.

Runners must complete at least one ≥ 100 km ultramarathon in the year before the WSER to qualify. The number of races completed by this sample was homogenous, but the time spent training and distance run during training in the 3 months leading up to the WSER were not homogenous. The wide range of training volumes offered the opportunity to investigate if training volume (training time and running distance) was associated with primary cardiovascular measures (systolic BP or cfPWV) at rest before the race. We did not find an association between these variables. These findings are related to the Extreme Exercise Hypothesis, but more work is needed to understand the components of exercise training that may be detrimental to health.

## Limitations

This investigation has strengths, but there are several limitations. As noted, BP could have been affected by altitude (Olympic Valley: ~ 1890 m), the supine position (Netea et al. 2003; Rhodes et al. 2011), national or international travel, circadian rhythm disruptions due to travel, or the general excitement before the race (60/71 athletes tested within 24 h of WSER start). We asked participants to refrain from food, caffeine, tobacco, and strenuous exercise, but we did not ask them to refrain from recreational or prescription medications/drugs, which may affect BP. Also, we did not have sufficient data to determine if there was an influence of time on caffeine or food consumption and the data we collected. Given these limitations, the BP status results should not be treated as a clinical diagnosis of hypertension. We emphasize that more work is needed to understand typical BP in this growing population. Since this was a field study, we could not achieve the same level of environmental control as a traditional clinical or laboratory setting. All measures were taken in a canopy tent from ~ 7:00 am to ~ 6:00 pm, which could not be perfectly temperature controlled. However, associations between brachial systolic BP and cfPWV with time of day, temperature, and relative humidity were *negligible* (*r* < 0.25). There is a potential selection bias with this unique population. Namely, adults who have poorer cardiovascular health may be unable to continue to participate in ultramarathon running, which would lead to adults with better health continuing to run. Follow-up studies are needed to determine age-related changes on an individual level in this population.

## Conclusion

We found a significant relation between age and central but not brachial systolic BP, and the age-related difference in brachial systolic BP was lower than what has been observed in the general population. There was a significant curvilinear difference in cfPWV with age, but most athletes had values below their age-predicted values. There was not a significant association between training volume leading up to the race and BP or cfPWV. Future work is needed to characterize typical BP among this population, because most work has been performed only days before the start of a race. Also, more work is needed with participant follow-ups to determine longitudinal changes in cardiovascular health in the context of training for running ultramarathons. Furthermore, more work is needed to understand potential sex differences in cardiovascular adaptations and how adopting ultra-endurance running as a young adult versus as a middle-aged or older adult may differ. Overall, these data suggest that training for an ultramarathon race may benefit age-related differences in BP and cfPWV.

## Supplementary Material

Supplement

**Supplementary Information** The online version contains supplementary material available at https://doi.org/10.1007/s00421-025-05924-w.

## Figures and Tables

**Table 1:** Participant characteristics

Demographics (*n* = 71)	Mean ± SD

Male/female	55/16
Race (count)	58 W; 5 AS; 1 AS + W; 1 AAN; 1 AAN + W; 0 B; 4 UK
Ethnicity (count)	2 HL, 69 NHL
Age (years)	46 ± 10
Age range (years)	26–69
Height (cm)	176 ± 8
Mass (kg)	70.5 ± 9.2
Body mass index (kg/m^2^)	22.6 ± 1.8
Running experience (*n* = 70)	**Median [IQR]**
Years of running experience (years)	16 [15]

*SD* standard deviation, *W* White, *AS* Asian, *AS + W* Asian and White, *AAN* American/Alaskan Native, *AAN + W* American/Alaskan Native and White, *B* Black, *UK* Unknown, *HL* Hispanic/Latinx, *NH*L non-Hispanic/Latinx, *IQR* interquartile range, *WSER* Western States Endurance Run

Mean ± SD represents normally distributed data (Shapiro–Wilk *P* >0.05); Median [IQR] represents non-normally distributed data (Shapiro–Wilk *P* <= 0.05)

**Table 2:** Medical history

Chronic conditions (n = 71)	Count (%)		

	Yes	No	Not anymore

Abnormal chest X-ray	0 (0.0)	70 (98.6)	1 (1.4)
Anemia	0 (0.0)	70 (98.6)	1 (1.4)
Angina pectoris (chest pain)	0 (0.0)	70 (98.6)	1 (1.4)
Asthma	2 (2.8)	63 (88.7)	6 (8.5)
Eating disorder	0 (0.0)	69 (97.2)	2 (2.8)
Heart murmur	0 (0.0)	70 (98.6)	1 (1.4)
High cholesterol	6 (8.5)	60 (84.5)	5 (7.0)
High triglycerides	1 (1.4)	70 (98.6)	0 (0.0)
Hypertension	4 (5.6)	66 (93.0)	1 (1.4)
Stroke/transient ischemic attack	0 (0.0)	70 (98.6)	1 (1.4)
Thyroid problems	1 (1.4)	70 (98.6)	0 (0.0)
Type 1 diabetes	1 (1.4)	70 (98.6)	0 (0.0)
Type 2 diabetes	0 (0.0)	71 (100)	0 (0.0)

Blood pressure and pulse wave analysis. Summary data for BP and pulse wave analysis are shown in Table 3

**Table 3:** Blood pressure and pulse wave analysis summary

Pulse wave analysis (*n* = 71)	Mean ± SD Median [IQR]

Central systolic BP (mmHg)	116 ± 8
Central mean BP (mmHg)	93 ± 7
Central diastolic BP (mmHg)	79 ± 7
Brachial systolic BP (mmHg)	129 ± 9
Brachial mean BP (mmHg)	95 ± 7
Brachial diastolic BP (mmHg)	78 ± 7
AP (mmHg)	8 ± 5
AIx (%)	20.5 [12.5]
AIx75 (%)	13.6 [14.2]

*SD* standard deviation, *IQR* interquartile range, *BP* blood pressure, *AP* augmentation pressure, *AIx* augmentation index, *AIx75* augmentation index normalized to a heart rate of 75 beats per minute; mean ± SD represents normally distributed data (Shapiro–Wilk *P* > 0.05); median [IQR] represents non-normally distributed data (Shapiro–Wilk *P* <= 0.05)

## Data Availability

Data are available upon reasonable request to the principal investigator after institutional data transfer approval.

## Abbreviations

AAN: American/Alaskan Native
AIx: Augmentation Index
AIx75: Augmentation Index Normalized to a Heart Rate of 75 bpm
AP: Augmentation Pressure
AS: Asian
AS + W: Asian and White
B: Black
BMI: Body Mass Index
BP: Blood Pressure
cfPWV: Carotid-to-Femoral Pulse Wave Velocity
CVD: Cardiovascular Disease
HL: Hispanic/Latinx
HR: Heart Rate
IQR: Interquartile Range
NHL: Non-Hispanic/Latinx
PP: Pulse Pressure
SD: Standard Deviation
UK: Unknown
W: White
WSER: Western States Endurance Run
