# Supplementary material for: Central blood pressure and arterial stiffness among ultramarathon runners across the lifespan
Source: Eur J Appl Physiol. 2025 Jul 30;126(1):529–39. doi: 10.1007/s00421-025-05924-w (PMC12356499; doi:10.1007/s00421-025-05924-w)
Supplement: Supplementary file 1 — Supplementary file1 (DOCX 28 KB) [file 421_2025_5924_MOESM1_ESM.docx]

Supplemental Table 1. Medications (last 3 months)

| **Reported medication use** | **Count (%)** |
| --- | --- |
| 0 medications | 46 (64.8) |
| 1 medication | 16 (22.5) |
| 2 medications | 6 (8.5) |
| 3 medications | 3 (4.2) |
| **Medications listed** (n = 71) | **Count (%)** |
| Antihistamine (allergy) | 7 (9.9) |
| Analgesic | 3 (4.2) |
| Antiacid | 3 (4.2) |
| Antibiotic | 1 (1.4) |
| Anticoagulant | 1 (1.4) |
| Antidepressant | 3 (4.2) |
| Antidiabetic | 1 (1.4) |
| Antifungal | 1 (1.4) |
| Antirheumatic | 1 (1.4) |
| Anti-viral | 1 (1.4) |
| Antihypertensive | 4 (5.6) |
| Anti-hypothyroid | 1 (1.4) |
| Anti-cholesterol (statin) | 1 (1.4) |
